# Japanese Encephalitis Virus Infected Human Monocyte-Derived Dendritic Cells Activate a Transcriptional Network Leading to an Antiviral Inflammatory Response

**DOI:** 10.3389/fimmu.2021.638694

**Published:** 2021-06-17

**Authors:** Shailendra Chauhan, Deepak Kumar Rathore, Shilpa Sachan, Sebastien Lacroix-Desmazes, Nimesh Gupta, Amit Awasthi, Sudhanshu Vrati, Manjula Kalia

**Affiliations:** ^1^ Translational Health Science & Technology Institute, Faridabad, India; ^2^ Vaccine Immunology Laboratory, National Institute of Immunology, New Delhi, India; ^3^ Institut National de la Santé et de la Recherche Médicale, Centre de Recherche des Cordeliers, Sorbonne Université, Université de Paris, Paris, France; ^4^ Regional Centre for Biotechnology, Faridabad, India

**Keywords:** Japanese encephalitis virus, flavivirus, monocyte-derived dendritic cell, innate immune response, NF-κB, Tregs, PPAR, lipid metabolism

## Abstract

A comprehensive understanding of the human immune response to virus infection is imperative for developing effective therapies, antivirals, and vaccines. Dendritic cells (DCs) are among the first cells to encounter the virus and are also key antigen-presenting cells that link the innate and adaptive immune system. In this study, we focus on the human immune response to the mosquito-borne Japanese encephalitis virus (JEV), which is the leading cause of virus-induced encephalitis in south-east Asia and has the potential to become a global pathogen. We describe the gene regulatory circuit of JEV infection in human monocyte-derived DCs (moDCs) along with its functional validation. We observe that JEV can productively infect human moDCs leading to robust transcriptional activation of the interferon and NF-κB-mediated antiviral and inflammatory pathways. This is accompanied with DC maturation and release of pro-inflammatory cytokines and chemokines TNFα, IL-6, IL-8, IL-12, MCP-1. and RANTES. JEV-infected moDCs activated T-regulatory cells (Tregs) in allogenic mixed lymphocyte reactions (MLR) as seen by upregulated FOXP3 mRNA expression, suggestive of a host response to reduce virus-induced immunopathology. The virus also downregulated transcripts involved in Peroxisome Proliferator Activated Receptor (PPAR) signalling and fatty acid metabolism pathways suggesting that changes in cellular metabolism play a crucial role in driving the DC maturation and antiviral responses. Collectively, our data describe and corroborate the human DC transcriptional network that is engaged upon JEV sensing.

## Introduction

The mosquito-borne flaviviruses such as Japanese encephalitis virus (JEV), Dengue virus (DENV), West Nile virus (WNV), and Zika virus (ZIKV) are a significant cause of disease globally, with regular epidemics reported from many countries. JEV is majorly observed in South-East Asia, and despite availability of vaccines, is a leading cause of encephalitis-related morbidity and mortality in these parts of the world. Close to 100,000 cases of Japanese encephalitis (JE) occur annually associated with a high case-fatality rate of 20-30%. Significant neurological and psychiatric sequelae persist in nearly 30-50% of the survivors (1, 2). Based on these data, JEV infection and its associated immune-pathologies and disease, continue to remain a major challenge to public health. The lack of effective antiviral therapies reinforces the need to understand how the human immune response to JEV infection is regulated.

JEV pathogenesis has been primarily explored through *in vitro* studies and animal models (3, 4). The disease manifests upon ineffective clearance of the virus from the periphery, which ultimately leads to breaches in the blood-brain barrier (BBB) through one or more of several potential mechanisms (5, 6). The peripheral immune response is thus critical for limiting neuroinvasion and determines the outcome of infection. Most infected individuals recover completely and develop robust protective immunity, as both an effective humoral and T cell response determines the clinical outcome of disease (7, 8).

Dendritic cells (DCs) prime T cells through antigen presentation and therefore are considered as linkers of the innate and adaptive immune responses. DCs play a key role in driving an adaptive immune response during virus infection. DC maturation, activation and the secretion of specific cytokines ultimately determine the quality of the T cell response (9). JEV cannot replicate in human erythrocytes, granulocytes or lymphocytes (10). Monocytes, macrophages and DCs in blood and tissue have been established as JEV permissive cells in both humans and mice (10–14). The C-type lectin receptor DC-specific intercellular adhesion molecule-3-grabbing non-integrin (DC-SIGN), has been shown to act as the JEV receptor on DCs, and facilitates its transmission to T cells *via* the virological synapse (15).

Studies in the mouse model have elucidated that JEV-infected bone-marrow-derived DCs (bmDCs) do not upregulate the surface expression of CD40 and MHC class II, and release both pro-inflammatory cytokines (IL-6, TNFα, IL-12) and the anti-inflammatory cytokine IL-10, resulting in inadequate CD8^+^ T cell priming and ineffective CTL responses (11, 16). Moreover, JEV could suppress *in vivo* cross-presentation of soluble and cell-associated antigens through the TLR2-MyD88 and p38 MAPK-signalling (17). Another study has shown that JEV infection of mouse bmDCs and spleen-derived DCs inhibited the expression of maturation markers and expanded CD4^+^Foxp3^+^Tregs (12).

JEV infection of human moDCs leads to the induction of maturation markers along with CD274/PD-L1. The PD-L1-PD1 axis was implicated in the expansion of Tregs *via* JEV-infected DCs (18). A recent study has shown that JEV infects human moDCs and induces TNF and INF-β (19). However, the primary human innate immune response to JEV remains incompletely characterized.

In the present study, we describe the transcriptional circuitry of human moDCs after JEV infection. We observe activation of the DC antiviral and inflammatory gene regulatory network. We further experimentally validate the resulting DC maturation program and inflammatory cytokine release. JEV-infected DCs generated Tregs in an allogenic response. Downregulation of lipid and fatty acid metabolic pathway transcripts highlighted the role of metabolism in controlling dendritic cell functions.

## Materials and Methods

### Ethics Statement

Peripheral blood mononuclear cells (PBMCs) were isolated from blood obtained from healthy volunteers after written informed consent. THSTI Human Ethics committee approved the protocol.

### Generation of Immature moDCs

An experimental system for the generation of human moDCs was established. PBMCs were isolated from whole blood using density gradient centrifugation (20). Primary human monocytes (CD14^+^) were isolated from PBMCs using CD14^+^ magnetic beads (130-050-201, Miltenyi Biotec) as per the manufacturer’s protocol. The purity of the monocyte prep (>95% CD14^+^cells) was established (**Figure S1**). The cells were differentiated *in vitro*, by incubating in Mo-DC differentiation medium (130-094-812, Miltenyi Biotec), and by day 5 immature DCs were obtained (CD14^-^ CD209^+^). The quality of the prep was confirmed through the moDCs differentiation inspector kit (130-093-567, Miltenyi Biotec) (**Figure S1**). Cell viability was examined using an automated cell counter (Countess™ II FL Automated Cell Counter, Invitrogen) through Trypan Blue exclusion.

### Cell Lines

Vero and C6/36 cell lines were obtained from National Centre for Cell Science, Pune. Vero cells were maintained in Minimal Eagle’s media (MEM) with 10% foetal bovine serum (FBS) and antibiotics. C6/36 cells were maintained in Leibovitz’s L-15 Medium.

### Virus Generation and Assays

JEV genotype 3 strain P20778 (AF080251) was used in this study (21). Virus was generated in C6/36 cell line and concentrated through PEG precipitation, followed by purification through sucrose-based density gradient ultracentrifugation. The moDCs were infected with purified JEV at 5 MOI for 1 h at 37°C in RPMI medium with 2% FBS. After 1 h of virus incubation, cells were washed twice with PBS and resuspended in complete medium. At the indicated time post-infection, cells were harvested for subsequent experimentation. qRT-PCR for JEV was performed as described previously (22). Virus titration was performed through focus forming unit (FFU) assays in Vero cells. Briefly, cells were grown in a 24-well plate, and 10-fold serially diluted virus stock was added for 1 h at 37°C with gentle rocking. After washing, cells were incubated with complete medium for 48 h, followed by immunostaining with flavivirus anti-E (4G2) primary antibody, and HRP-conjugated secondary antibody. Foci were rendered visible by treatment with true blue peroxide substrate for 30 min. The virus titre was calculated by the following formula: Virus titre (ffu/ml) = Average count of foci/Volume of infection (ml) x Dilution factor.

### Quantification of mRNA

Expression levels of various genes were evaluated using qRT-PCR assays. Total RNA extraction was performed using Trizol reagent. The cDNA was prepared using random hexamers with the GoScript™ Reverse Transcription System (Promega). The samples were analysed in technical triplicates by SYBR green PCR (SYBR premix Ex Taq, Takara) on a QuantStudio 6 Flex Real-time PCR machine (Applied Biosystems). Relative expression of each gene during JEV infection was calculated using the Ct method with mock infection as the reference and GAPDH as an internal control. Primer sequences of all the genes tested in the study are given in **Table S1**.

### Co-Stimulatory Protein Levels

Surface levels of DC maturation and other markers (CD80, CD83, CD86, CD209, CD274 and HLA-DR) were assessed by flow cytometry. The fluorochrome-conjugated monoclonal antibody staining was performed as per the manufacture’s protocol. Analysis was done on BD Biosciences FACSCanto II flow cytometer. Data was analysed using FlowJo (TreeStar). The antibodies used in the study are listed in **Table S2**.

### Mixed Lymphocyte Reaction

Naïve CD4^+^ T cells were isolated from PBMCs obtained from healthy donor peripheral blood using the CD4^+^ T cell isolation kit (130-045-101, Miltenyi Biotech). Immature moDCs were mock/JEV infected (5MOI, 24 h), and washed thoroughly before co-culture. In a 96-well U bottom plate, mock/JEV-infected moDCs were co-cultured with CD4^+^ naïve T cells (1:10 ratio) in RPMI supplemented with 10% human serum for 4 days (18). mRNA levels of the T-subset specific transcription factors T-BET, GATA3, FOXP3 and RORγt was analysed through qRT-PCR.

### Cytometric Bead Array (CBA)

CBA was performed to quantitatively measure the cytokine level of IL-6, IL-8, IL-10, IL-12, MCP-1, RANTES, IFNγ and TNFα in JEV-infected moDCs using the CBA Flex kit from BD biosciences (**Table S3**). Samples were prepared according to manufacturer’s protocol and acquired on BD Biosciences FACSCanto II flow cytometer. Analysis was performed using CBA software FCAP array™ v3.0.1. The quantity of the cytokines detected in the samples was measured against the standard curve obtained from defined concentration of protein provided in the flex set kit (**Figure S2**).

### RNA Sequencing and Analysis

moDCs were generated from three donors and were mock/JEV infected at 5 MOI for 24 h. Cell pellets stored in Trizol were sent for further processing (RNA extraction, rRNA depletion, sequencing library preparation) and RNA sequencing to the National Institute of Biomedical Genomics, Kalyani, India. Paired-end sequencing (2 x 100 bp) was performed on Illumina HiSeq 2500 System. Reads that passed quality thresholds (Phred Score <30; FastQC, Version 0.11.9 followed by adapter removal through Trim Galore, Version 0.6.5) were used for further analyses and were mapped to the latest stable version of the human reference genome GH38 (GRCh38.p5, Ensembl) using Bowtie2 and Tophat 2.1.1 (23). The expression of the assembled transcriptomes was estimated using Cufflinks 2.2.1 (24, 25). Computation of normalized gene and transcript expression profiles for each sample was performed. The FPKM (Fragments Per Kilobases per Million fragments) method was used followed by log_2_ transformation of the value. The gene-level differential expression between mock- and JEV-infected conditions were estimated using the log_2_-transformed FPKM. The uncorrected p-value of the test statistic, and the FDR-adjusted p-value of the test statistic (q-value), were also estimated during the process of identifying differentially expressed genes (DEGs). Any gene with a p-value greater than the FDR, after Benjamini-Hochberg correction for multiple-testing, was deemed significantly differentially expressed in the JEV-infected condition as compared to the mock. Several graphical representations were also created using the R package CummeRbund [V2.7.2.].

### Statistical Analysis

All statistical analyses were performed using GraphPad Prism, Version 8.1.2 (332) software. Statistical significance was determined by *p*-value of <0.05 using Wilcoxon matched-pairs signed rank test.

## Results

### JEV Replication in Human moDCs

**Figure 1:**
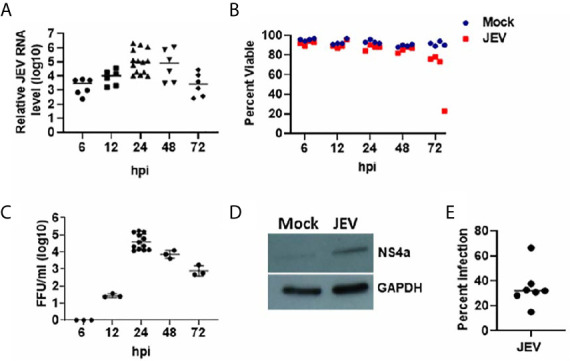
JEV replication kinetics in human moDCs. **(A–C)** Immature moDCs from multiple donors were mock/JEV infected at 5MOI, and samples were harvested at the indicated time post-infection (hpi: hour post-infection) for analysis. **(A)** Relative JEV RNA levels for each donor with mean, normalized to mock infection (*n* = 6 to 14 donors). **(B)** Cell viability of mock/JEV infected moDCs for *n* = 4 donors. **(C)** Virus titres in the culture supernatant for each donor with mean (*n* = 3 to 12 donors). **(D)** Western blot showing expression of JEV NS4a protein in cell lysate of JEV infected moDCs at 24 hpi. GAPDH blot serves as a loading control. **(E)** Percentage of JEV infected moDCs at 24 hpi as estimated by 4G2 antibody staining through flow cytometry. Data along with mean are shown for *n* = 7 donors.

Monocytes isolated from the blood of healthy individuals were differentiated into immature DCs that showed high surface expression of CD209 (**Figure S1**). These were infected with JEV at 5 MOI, and replication kinetics of JEV within the moDCs was monitored by measuring relative expression of viral RNA levels in cells and virus titres in the culture supernatant, across multiple donors. As indicated, JEV replication progressed rapidly 6 h post-infection (hpi) and plateaued between 24 and 48 hpi (**Figure 1A**). Infected moDCs remained healthy up till 48 hpi, after which a decline in the viability of JEV infected-moDCs was observed (**Figure 1B**). Consistently, virus titres in the culture supernatant of infected moDCs also peaked at 24 hpi (**Figure 1C**). Active virus replication at 24 hpi was also validated by detection of NS4a protein through Western blotting (**Figure 1D**). Flow cytometry analysis using the pan-flavivirus 4G2 antibody estimated the percentage of JEV-infected cells to be in the range of 15-66% at 24 hpi (**Figure 1E**). In comparison, JEV infection at 1 MOI resulted in a very low number of infected cells and significantly delayed replication kinetics (data not shown). Hence JEV infection at 5 MOI for 24 h was chosen for all further analyses. Collectively, these data established that human moDCs support productive JEV replication.

### Transcriptome Analysis of Host Response to JEV Infection

**Figure 2:**
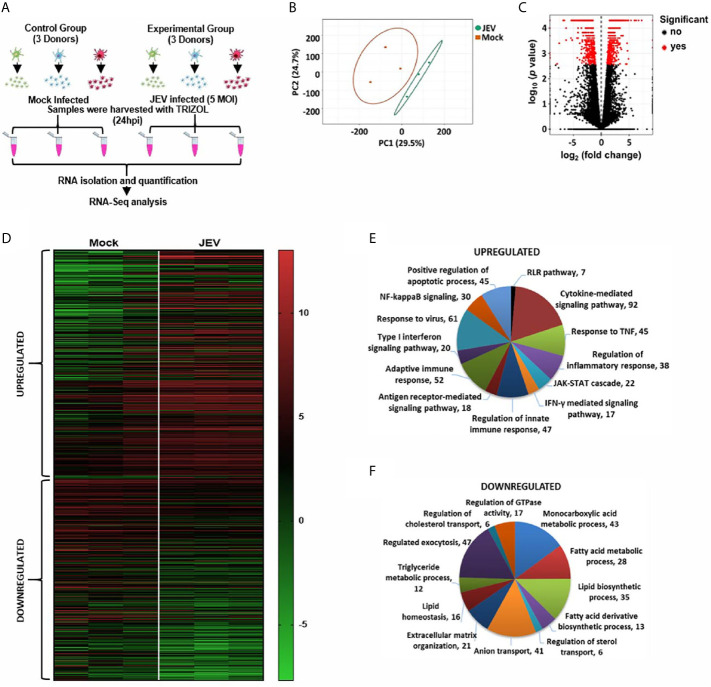
RNA-seq analysis of JEV infected moDCs. **(A)** Schematic representing the work plan for RNA-seq analysis. moDCs from three healthy donors were mock/JEV infected (5 MOI, 24 h), and RNA-seq analysis was performed. **(B)** The normalized raw intensities of all samples was used to generate the Principal-component analysis (PCA) plot using ClustVis server. **(C)** Volcano plot showing genes detected by RNAseq. Differentially expressed genes (DEGs) are marked as red dots and were identified on the basis of log_2_ fold-change ≥2 (FDR-adjusted *p* < 0.05) **(D)** Heatmap showing hierarchical clustering of DEGs between mock and JEV-infected condition in the three donors. **(E, F)** Gene Ontology (GO) enrichment analysis of upregulated and downregulated genes in JEV *vs* mock condition was performed using Metascape to study biological processes.

To gain insights into the transcriptional programming of JEV-infected moDCs, we performed an RNA-seq analysis of mock- and JEV-infected moDCs from three donors (**Figure 2A**). Principal-component analysis (PCA) was performed to assess the overall similarity of the expressed genes from each sample within these two groups, and it showed independent clustering of the mock and infected samples (**Figure 2B**). Differentially expressed genes (DEGs) were identified on the basis of log_2_ fold-change ≥ 2 (FDR-adjusted p < 0.05) (**Figures 2C, D**). Pathway enrichment analysis of the DEGs was performed on the basis of biological processes, molecular functions and cellular compartments (**Figures 2E, F**; **S3**). The key functional networks observed to be upregulated were those related to activation of the innate immune pathway (interferon/JAK-STAT signalling); cytokine secretion and signalling; NF-κB activation and inflammation; and apoptosis (**Figure 2E**). The significantly downregulated transcripts were majorly involved in monocarboxylic acid, fatty acid and lipid metabolic processes, exocytosis, sterol transport and extracellular matrix organization (**Figure 2F**).

### JEV-Infected Human moDCs Upregulate Maturation Markers and CD274

**Figure 3:**
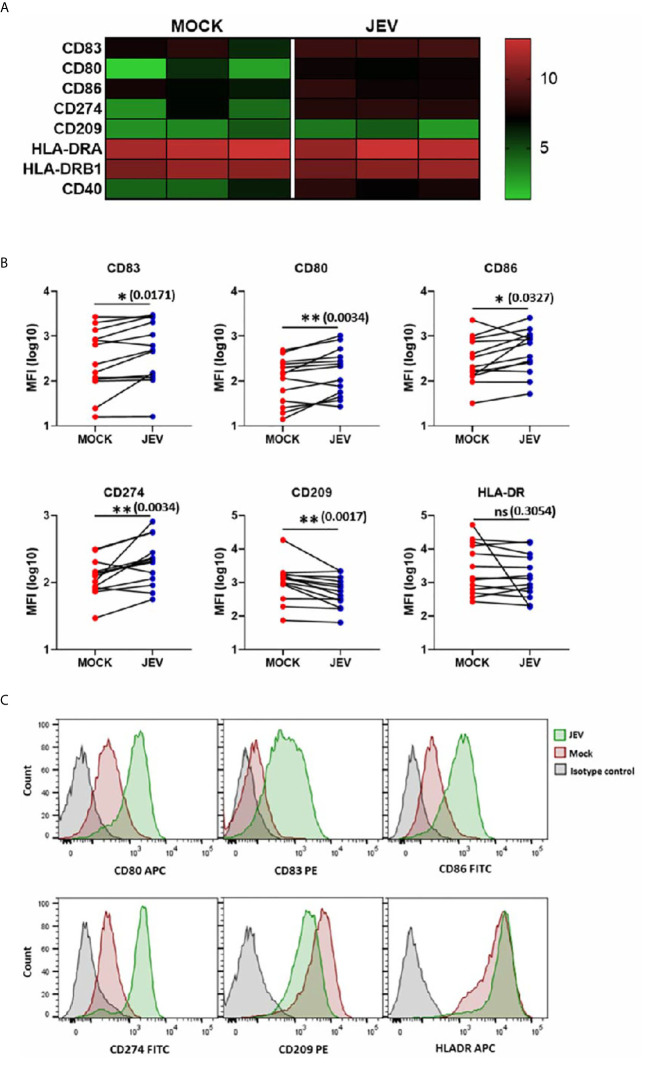
Upregulation of maturation markers in JEV infected moDCs. **(A)** Heatmap showing transcriptional upregulation of DC maturation and other co-stimulatory markers in mock *vs* JEV-infected condition in the three donors. **(B, C)** The cell surface expression of DC maturation and co-stimulatory markers was quantified by flow cytometry in mock/(5 MOI) JEV-infected moDCs at 24 hpi. Data for each donor is shown as median fluorescence intensity (MFI) with the mean (*n* = 13 donors). *p < 0.05; **p < 0.01 (Wilcoxon matched-pairs signed-rank test). **(C)** Representative flow cytometry profile of one donor. Ns, not significant.

**Figure 4:**
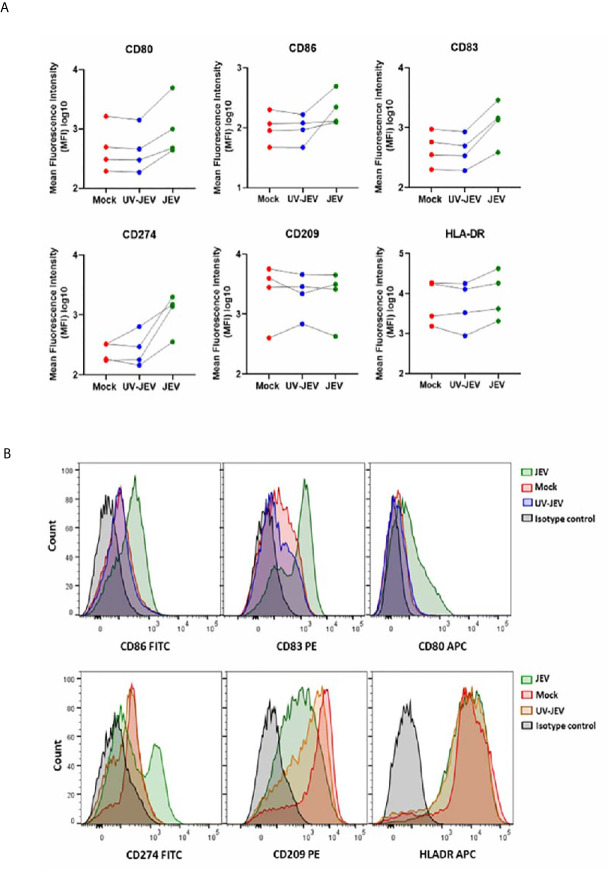
UV-inactivated JEV does not lead to DC maturation. **(A)** The cell surface expression of DC maturation and co-stimulatory markers was quantified by flow cytometry in mock/(5MOI) UV-inactivated JEV/(5MOI) JEV infected, moDCs at 24 hpi. Data for each donor is shown as median fluorescence intensity (MFI) with the mean (*n* = 4 donors). **(B)** Representative flow cytometry profile of the experiment.

Upon activation, DCs undergo a maturation program that is marked by an increased surface expression of co-stimulatory molecules, which enhances their ability to prime T-cell responses (26). We first tested DC maturation signatures in our transcriptome data, and observed upregulation of CD80, CD86, CD83 and CD40 expression (**Figure 3A**). In concordance with our RNA-seq data, we observed a modest but significant upregulation of CD80, CD83, and CD86 in JEV-infected moDCs (**Figures 3B, C**). CD209/DC-SIGN showed downregulation at both transcriptional and protein levels, while HLA-DR showed no significant change (**Figures 3A, B**). As reported earlier (18), significant upregulation of CD274 (PD-L1) ranging from 14-75% was also observed (**Figures 3A, B**). This indicates that JEV-infected moDCs undergo maturation. In contrast, UV-inactivated JEV did not increase the surface expression of CD80, CD83 CD86 and CD274 (**Figure 4**), suggesting that productive replication of JEV in moDCs is essential to activate the maturation program.

### JEV Infection Leads to Upregulation of Innate Immune and Inflammatory Signalling

**Figure 5:**
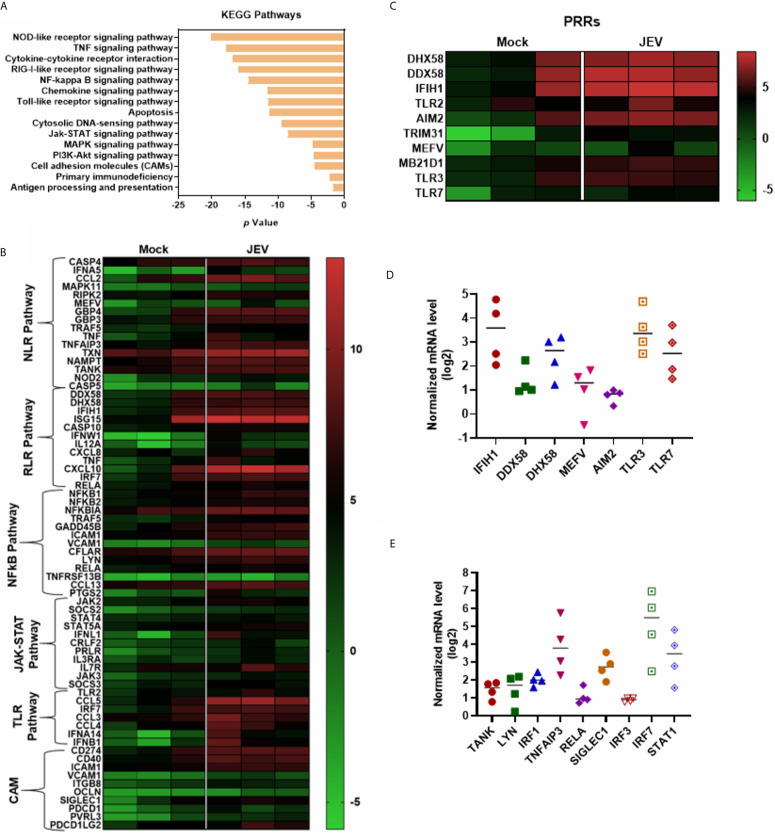
Activation of innate immune and inflammatory responses in JEV-infected moDCs. **(A)** KEGG pathway enrichment analysis of the upregulated immune-related significant DEGs in JEV-infected moDCs. **(B, C)** Heatmap showing DEGs mapped to various innate immune and inflammatory pathways **(B)**, and Pathogen recognition receptors (PRRs) **(C)**, between mock and JEV-infected condition across the three donors. **(D, E)** Relative mRNA levels of selected PPRs **(D)** and innate immune/inflammatory regulators **(E)** was analysed in JEV-infected (5 MOI, 24 h) moDCs from four donors by qRT-PCR. Value from each donor along with the mean shown as log_2_ expression normalized to mock infection.

**Figure 6:**
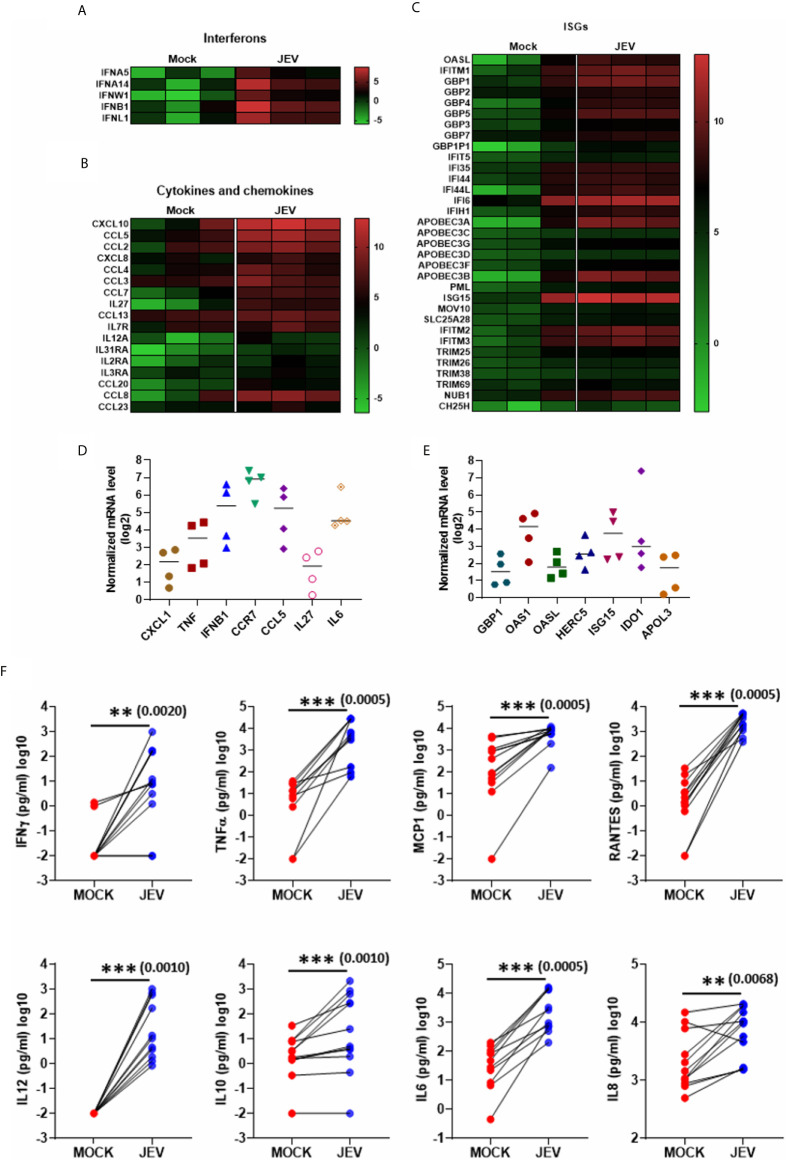
Activation of interferons, cytokines, chemokines and Interferon stimulated genes (ISGs) in JEV-infected moDCs. **(A–C)** Heatmap showing DEGs coding for interferons **(A)**, cytokines and chemokines **(B)**, and ISGs **(C)**, between mock and JEV infected condition from three donors. **(D, E)** Relative mRNA levels of selected cytokines/chemokines **(D)** and ISGs **(E)** was analysed in JEV-infected (5 MOI, 24 h) moDCs from four donors. Value from each donor along with the mean shown as log_2_ expression normalized to mock infection. **(F)** Secretion of various cytokines and chemokines was analysed by multiplex bead array following JEV infection of moDCs (5 MOI, 24 h). Data for 12 donors is shown along with the mean. **p < 0.01; ***p < 0.001 (Wilcoxon matched-pairs signed-rank test).

KEGG pathway analysis of the upregulated DEGs highlighted the following signalling pathways: NOD-like receptor, cytokine receptor, TNF, RIG-I & Toll-like receptor, Jak-STAT and NF-κB, showing a robust activation of innate immune signalling (**Figures 5A, B**). Most of the critical PRRs such as RIG-I (DDX58), MDA-5 (IFIH1), LPG2 (DHX58), Pyrin (MEFV), AIM2, TLR2, TLR3 and TLR7 were upregulated in the transcriptome data (**Figure 5C**), and their activation was substantiated in independent samples by qRT-PCR (**Figure 5D**). Upregulation of several of the crucial transcription factors (TF) and related genes involved in innate immune and inflammatory signalling were also validated (**Figure 5E**). JEV infection also led to robust activation of interferons, cytokines and chemokines, and a diverse panel of interferon-stimulated genes (ISGs) (**Figures 6A–C**). Activation of a select group of cytokines, chemokines and ISGs was also verified independently by qRT-PCR (**Figures 6D, E**). A crucial aspect of DC activation is the release of pro-inflammatory mediators that modulate the immune response. We observed that JEV-infected moDCs secreted significantly high amounts of TNFα, MCP-1, RANTES and the cytokines IL-12, IL-6 and IL-8 (**Figure 6F**). In addition to these proinflammatory cytokines, the key regulatory cytokine IL-10 was also secreted (**Figure 6F**). Collectively our data indicate that JEV infection of moDCs leads to their maturation and robust activation of innate and inflammatory response, along with the secretion of an array of inflammatory cytokines.

### JEV Infection Upregulates Treg Cells

**Figure 7:**
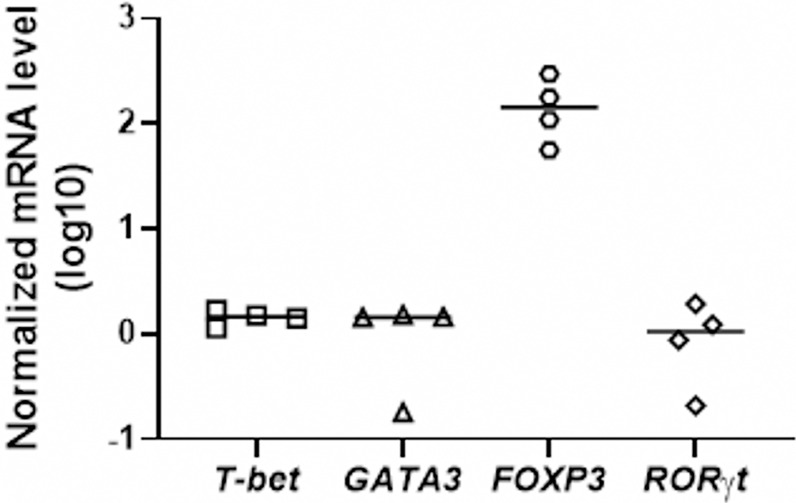
JEV-infected moDCs lead to expansion of Tregs in mixed lymphocyte reactions. CD4^+^ T cells were co-cultured with mock/JEV infected moDCs at 10:1 (T:DC) ratio in an allogenic co-culture assay for 4 days. mRNA levels of T-subset specific transcription factors T-BET, GATA3, FOXP3 and RORγt were analysed through qRT-PCR. Plotted are values and mean from 4 independent experiments shown as log_10_ expression normalized to mock-treated moDCs.

We next checked the functional capacity of JEV-infected moDCs to induce T-cell activation in allogeneic mixed lymphocyte reactions. To understand which effector and/or regulatory T-cell subsets are induced by JEV-infected moDCs, specific transcriptional factors of Th1, Th2, Th17 and Tregs were tested in the moDC-T cell co-culture experiments. A significant upregulation of FOXP3 mRNA expression was observed in moDCs-T cells co-culture assays, suggesting the induction of Treg population (**Figure 7**).

### Downregulation of PPAR Signalling and Fatty Acid Metabolism Genes in JEV-Infected moDCs

**Figure 8:**
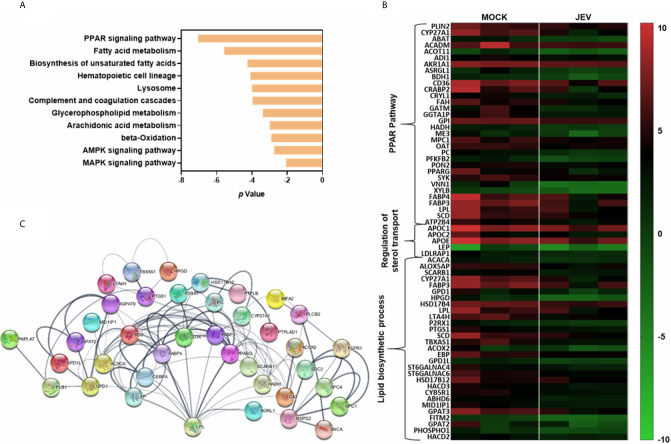
JEV-infected moDCs transcriptionally downregulate PPAR/lipid and fatty acid metabolism pathways. **(A)** Significantly downregulated DEGs during JEV infection were subjected to KEGG pathway enrichment. **(B)** Heatmap showing the downregulated DEGs encoding for proteins implicated in PPAR, regulation of sterol transport and lipid biosynthesis process pathways. **(C)** A functional gene association network for DEGs involved in lipid biosynthetic processes was generated using STING 11.0. The line thickness (-) indicates the strength of data support.

Metabolic changes through nuclear receptor family of transcription factors such as PPARγ and liver X receptor (LXR) have been shown to influence innate immune and inflammatory pathways in macrophages and DCs (27, 28). Activation of DCs by TLR agonists results in enhanced glycolysis and metabolic conversion. Downregulation of macrophage PPARγ signalling and sterol metabolic pathways has been reported earlier in the context of virus infections (29, 30). KEGG analysis of the significantly downregulated DEGs in our data highlighted the PPAR/lipid and fatty acid metabolism pathways suggesting global changes in the DC metabolism during JEV infection (**Figure 8A**). Critical transcripts of PPAR/lipid biosynthetic pathway (LPL, PPARG, FABP3, FABP4, SCD), and regulation of sterol transport were found to be suppressed in infected moDCs suggesting that downmodulation of lipid metabolism is likely to be intimately linked to an inflammatory response during JEV infection (**Figures 8B, C**).

## Discussion

The expanding threat of zoonotic virus infections to mankind highlights the necessity and urgency of biomedical research-driven health-care solutions. Defining the molecular signatures of the human immune response during pathogen infection is crucial to understand the disease process and for designing effective therapies.

The pathogenesis of JEV infection is a combination of direct virus-induced neuronal cell death and the host neuroinflammatory response (31). The magnitude and phenotype of the immune response mounted during JEV infection can restrict virus replication before and after the BBB is breached. The protective effect of neutralizing antibodies is well documented in both human and animal studies (8, 32, 33), and is one avenue for development of therapeutics (34). Further, an efficient adaptive immune response by CD4^+^ T-cells is involved in effective antibody production and prevention of virus entry in the CNS (32, 35). The quality of the CD4^+^ T-cell response was found to be a crucial factor most strongly associated with complete recovery from JE (8). A recent study in the mouse model of JE has also shown a crucial role of CD4^+^ T-cells in protective immunity and humoral response, which is augmented by the vaccine primed CD8^+^ T-cells (36).

DCs play a crucial role in the regulation of adaptive immune response to virus infection *via* regulation of T-cell priming (37). The aim of this study was to define the molecular signatures of human DC responses following JEV infection in moDC from healthy donors. We observe on an average 30% JEV infection in the human moDCs. Thus, in addition to direct infection, it is possible that the effect of secreted soluble factors (cytokines/chemokines) on the uninfected cells also contribute to the observed transcriptional changes. We observe strong activation of innate immune responses and an antiviral inflammatory program resulting in DC maturation. This was however accompanied by Treg expansion, suggesting a reduction of effector T-cell responses.

The transcriptional signatures of DENV-2, WNV and ZIKV infections in human moDCs have been characterized (38–40). DENV-2-infected moDCs undergo maturation and activate a robust antiviral and inflammatory program that was shown to be dependent on the infection-generated ROS and Nrf2 transcription factor (39). Another study showed high levels of cytokine secretion by DENV-2-infected moDCs, but poor T-cell priming (41). ZIKV-infected moDCs upregulated host antiviral proteins and type I IFN transcriptionally, but showed minimal up-regulation of maturation markers and poor cytokine secretion (38). Several strains of ZIKV were also shown to inhibit STAT1 and STAT2 phosphorylation. Similarly, while WNV-induced transcription of antiviral effector genes, transcription and secretion of proinflammatory cytokines was blocked (40). WNV-infected moDCs also underwent minimal maturation and poorly stimulated CD4 and CD8 T-cells, suggesting compromised T-cell immunity. STAT5 was identified as a key regulator of DC activation and immune response and was specifically shown to be antagonised by WNV and ZIKV. However, the four serotypes of DENV and the Yellow fever virus 17D vaccine strain (YFV-17D) did not negatively affect STAT5 phosphorylation (42). The YFV-17D lead to efficient activation of innate immune sensors, DC maturation and secretion of proinflammatory cytokines (43).

While the human DC responses to the major flaviviruses is well documented as described above, only few studies have examined this interaction in the context of JEV previously (18, 19). It was shown that JEV-infected moDCs undergo a maturation program along with upregulation of PD-L1 and expansion of Tregs (18). Here we expand the field further and show that JEV is proficient in activating the innate immune and inflammatory response pathways in human moDCs, as seen by transcriptional upregulation of several innate immune sensors, interferons, cytokines, chemokines and ISGs. This was accompanied by DC maturation and robust secretion of several proinflammatory cytokines and chemokines. This suggests that unlike WNV and ZIKV, JEV-infected human moDCs activate an antiviral program.

The DC immunophenotype and functional specification is also intimately linked to cellular metabolism (44–46). Our transcriptomic data showed a clear downregulation of PPAR/lipid and sterol metabolism pathways during JEV infection. An inverse relationship between lipid metabolism and innate immune responses has been shown in several studies (27, 45, 47). PPARγ, a lipid-activated transcription factor associated with lipid metabolism and adipocyte development, has been shown to modulate inflammatory responses in macrophages and DCs (48, 49). PPARγ agonists repress NF-κB, AP-1, STATs and IRF3 exerting a strong anti-inflammatory effect (28, 50, 51). PPARγ activation following TLR stimulation downregulated DC activation markers and reduced secretion of T cell stimulatory cytokines (29). Transcriptionally PPARγ was observed to be downregulated in alveolar macrophages following Influenza A virus infection in an INF-dependent manner (30). INFγ and IFNβ secretion during virus infection leads to downmodulation of sterol metabolic pathways and reduction in metabolic output in macrophages (52). Our study suggests that a similar downmodulation of lipid/sterol metabolism in moDCs may be linked to an effective immune response.

As has been reported previously (18), we observe that the functional outcome of JEV infection in moDCs derived from healthy donors is Treg induction. Tregs have been shown to exert a beneficial effect in acute viral infections by maintaining an equilibrium between pathogen clearance and excessive inflammation related pathologies. Higher Tregs are protective against WNV, HSV and Coronavirus-induced encephalitis (53–55), while reduced Tregs have been reported in neuroinvasive WNV infections and in Covid-19 hospitalized patients (54, 56, 57). Thus, the observed expansion of Tregs by JEV-infected moDCs is likely to favour the virus at the early stages of infection by reducing effector T-cell responses, but serves as a protective mechanism for reducing virus-induced inflammatory damage later during infection.

Most people who get infected with JEV are asymptomatic and disease develops in less than 1% of cases. Thus, in most healthy individuals the virus is likely to be efficiently targeted and cleared through an efficient DC response. The immunological signatures of different clinical outcomes of JE infection remain to be understood. A comparative transcriptional study of moDCs from naturally-infected ZIKV patients and *in-vitro*-infected moDCs from healthy donors showed a significant downregulation of antiviral innate immune sensors and ISGs in patient samples (58). A similar comparative study with JE patient samples will define the likely causes of disease progression. It could also be possible that JE disease susceptibility has an underlying genetic cause. Further studies with other JEV strains/genotypes and patient samples are essential to gain a complete functional understanding of the JEV-DC interaction.

## Data Availability Statement

The datasets presented in this study can be found in online repositories. The names of the repository/repositories and accession number(s) can be found below: (https://www.ncbi.nlm.nih.gov/), GSE160178.

## Ethics Statement

The studies involving human participants were reviewed and approved by THSTI Human Ethics committee. The patients/ participants provided their written informed consent to participate in this study.

## Author Contributions

SC performed the experiments and analysed the data. DR helped with flow cytometry and analysed the data. SS helped with methodology. SL-D, NG, MK, and SV conceptualised the project, acquired funding, and designed experiments. NG, MK, SV, and AA supervised the project and provided resources. SC and MK wrote the initial draft. DR, SL-D, NG, MK, AA, and SV reviewed and edited the final manuscript. All authors contributed to the article and approved the submitted version.

## Funding

This work was supported by a grant from the Indo-French Center for Promotion of Advanced Research (CEFIPRA, Reference No. 5103–3), and from DBT intra-mural funds to THSTI and RCB. Further support was provided from SERB grant (ECR/2016/001814), DST, Government of India (to NG). SC is supported by a DBT-SRF fellowship.

## Conflict of Interest

The authors declare that the research was conducted in the absence of any commercial or financial relationships that could be construed as a potential conflict of interest.
