# Supplementary material for: Japanese Encephalitis Virus Infected Human Monocyte-Derived Dendritic Cells Activate a Transcriptional Network Leading to an Antiviral Inflammatory Response
Source: Front Immunol. 2021 Jun 17;12:638694. doi: 10.3389/fimmu.2021.638694 (PMC8247639; doi:10.3389/fimmu.2021.638694)
Supplement: Supplementary file 1 [file DataSheet_1.docx]

**Supplementary Figures**

**

**

**Figure S1. Isolation of CD14^+^ monocytes and *in vitro* differentiation into DCs.** (A) Representative flow cytometry profile of primary human monocytes (CD14^+^) isolated from PBMCs using CD14^+^ magnetic beads. (B) Flow cytometry profile of isolated CD14^+^ monocytes (day 1), and *in vitro* differentiated immature DCs (day 5), stained with CD14 and CD209 antibodies. (C) CD14^+^ monocytes (day1) and immature dendritic cells (day5) were stained using CD14, CD209, and CD83 antibodies to check for the quality of monocyte isolation and DC differentiation. Data along with mean are shown for (*n*= 6-10 donors).

**
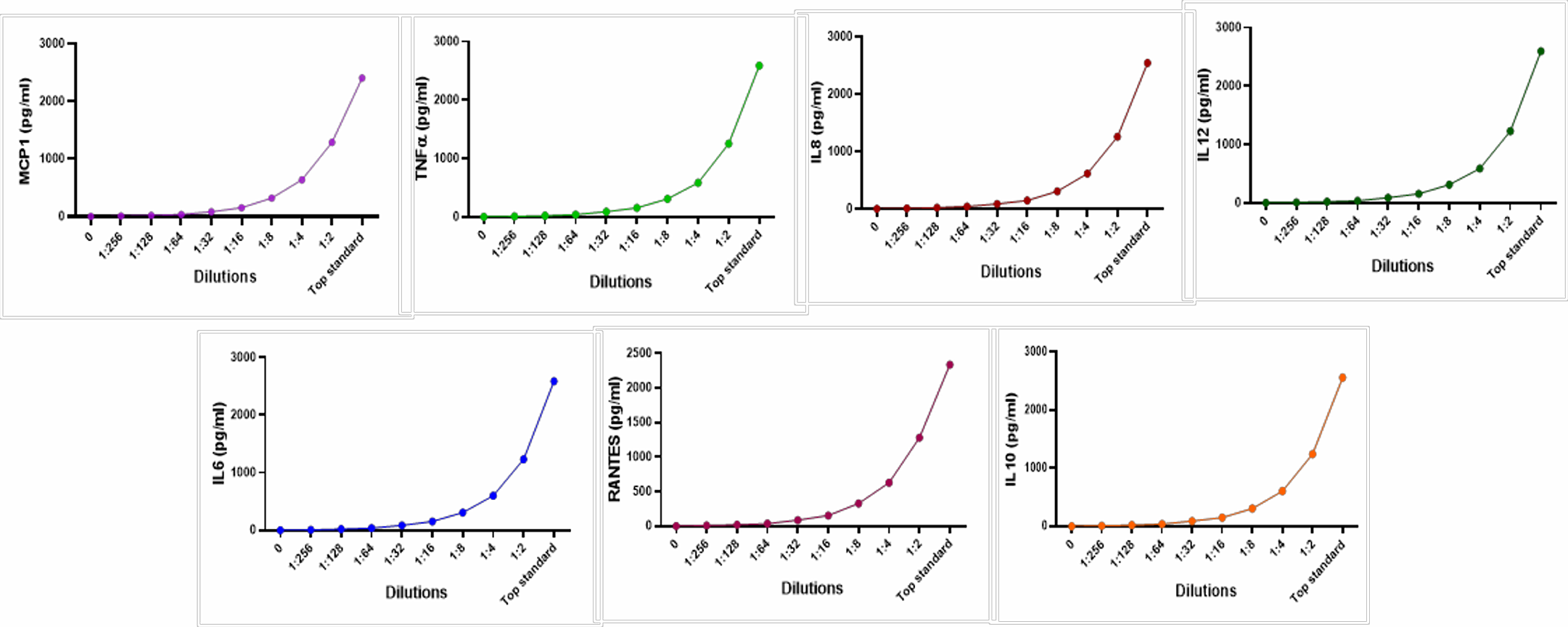
**

**Figure S2. Standard curves for CBA analysis of cytokines and chemokines secreted from JEV infected moDCs**. Analysis was performed using CBA software FCAP Array^TM^ v3.0.1

**
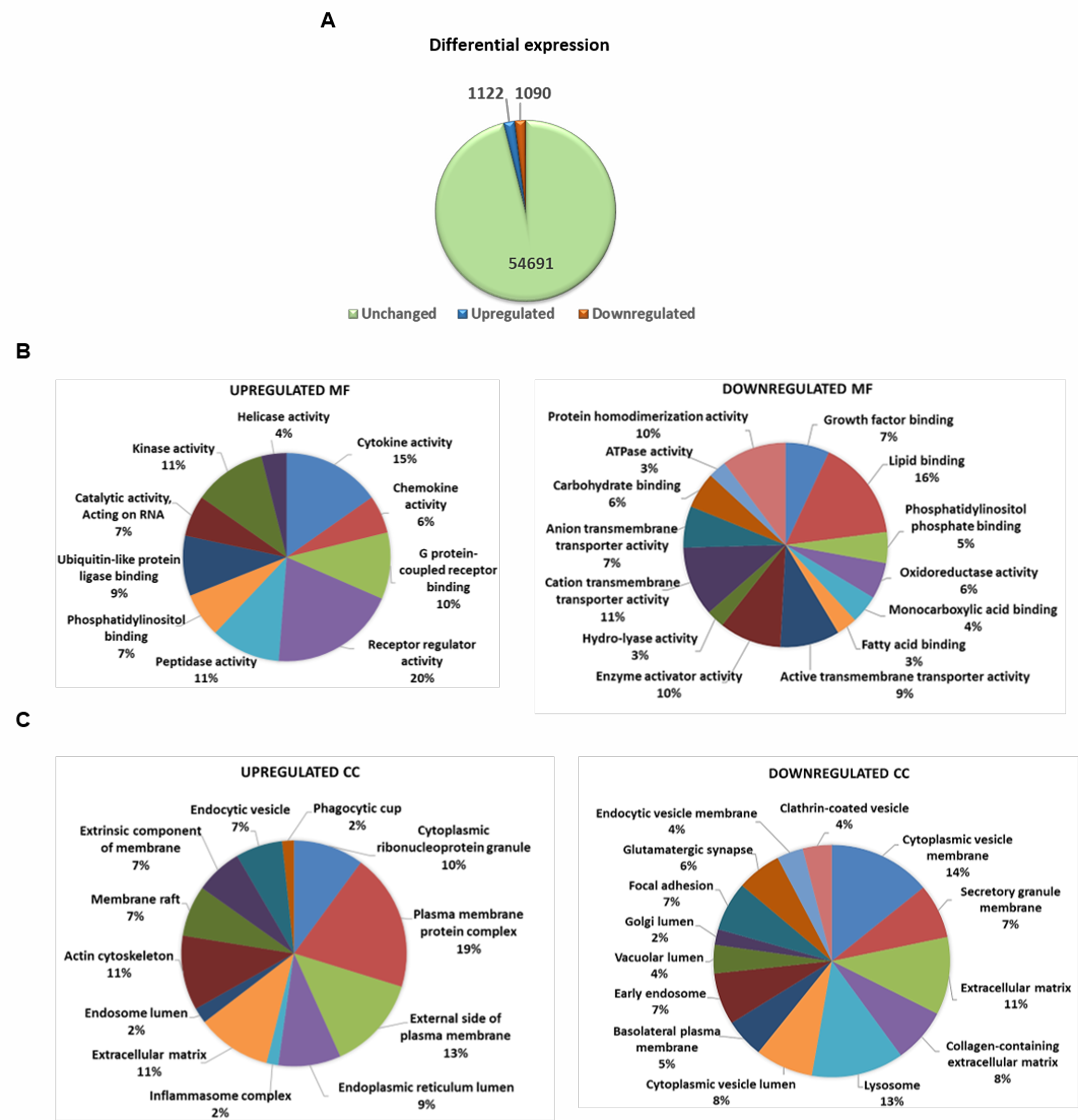
**

**Figure S3. RNA seq data analysis of JEV infected moDCs.** (A) Pie chart displaying the number of unchanged, and DEGs in the RNA seq analysis (log_2_ fold-change ≥ 2; FDR-adjusted *p* < 0.05). (B-C) Gene Ontology (GO) enrichment analysis of upregulated and downregulated genes in JEV vs mock condition was performed using Metascape to study molecular functions (MF) (B), and cellular compartments (CC), (C).

**Table S1: Primers sequences of genes tested in the study**

| **Gene name (human)** | **Primer Sequence (5’-3’)** |
| --- | --- |
| *Aim2-*F | CCTTCGTCCTTCCCTGACTT |
| *Aim2-*R | ATGGCCATTCTAGACCCTCG |
| *Apol3-*F | GGCACCACAATCCAAACCAT |
| *Apol3-*R | ATTGGGAAGGGAGTGAGTGG |
| *Ccl5-*F | CAGAAGGAGGAAGTCAGGCA |
| *Ccl5-*R | CTGTCCCTCTCTCTTTGGCA |
| *Ccr7-*F | CTTCGGTGTCCACTTTTGCA |
| *Ccr7-*R | CTTGCTGATGAGAAGGACGC |
| *Cxcl10-*F | CACAACCCAATCTGAAGCCA |
| *Cxcl10-*R | GGAAGTGATGGGAGAGGCAG |
| *Ddx58/RIG-I*-F | AGTGGGGTGAGGAGGAAATG |
| *Ddx58/RIG-I*-R | CAACACAACTCAGGCTTCCC |
| *Dhx58*-F | CACGTGCAGAAGATAAGCCC |
| *Dhx58*-R | AGTGGGAGGGGTAAGCTTTC |
| *Gapdh*-F | CGTCCCGTAGACAAAATGGT |
| *Gapdh*-R | TTGATGGCAACAATCTCCAC |
| *Gbp1-*F | AAGGCTCCTAACCCTCACAC |
| *Gbp1-*R | GTCTAAGCTGCCACACCTTG |
| *Herc5*-F | TTTTCTCTCTGGGTCGCACT |
| *Herc5*-R | GAGCTGAAAGGAGGAAGGGT |
| *Ido1-*F | TGATCTTGGACAATGGGCCT |
| *Ido1-*R | ACTGCAGTCTCCATCACGAA |
| *Ifih1/ MDA5*-F | TGCCTGTCCCACCATTGTAT |
| *Ifih1/ MDA5*-R | TCATCAGCCCAAGTGCCTTA |
| *Ifnb1-*F | CAGGTAGTAGGCGACACTGT |
| *Ifnb1-*R | TCAATTGCCACAGGAGCTTC |
| *Il27-*F | ACCCCACTCTGCCTTAAGTC |
| *Il27-*R | GGGCTCTGCTCTTCGAAATG |
| *Irf1*-F | GGGACACTAGGAAGGCAGTT |
| *Irf1-*R | GACTCAGCCTCTCAAACCCT |
| *Isg15-*F | GCCAATTTTCGTCTCCCTCC |
| *Isg15-*R | AGGGCTCGTTACTAGAAGGC |
| *JEV-*F | AGAGCACCAAGGGAATGAAATAGT |
| *JEV-*R | AATAAGTTGTAGTTGGGCACTCTG |
| *Lyn*-F | CCCCTCTTGAGCAGAAGTGA |
| *Lyn*-R | GTTCTGACACATGCCGATCC |
| *Mefv*-F | TTTCAGACATAGGCCACCGT |
| *Mefv*-R | GCCACCATGCCTAGCCTATA |
| *Oasl-*F | GGGCAGAGTCCTAGGTGTTT |
| *Oasl*-R | TCCCAGCACTCTCTTCCTTG |
| *RelA*-F | AGGTGTGGCTAGAACTGGAC |
| *RelA*-R | AGGAGAGAGACAGAGAGGCA |
| *Tank-*F | GCCATAACTGAGTCTGCTGC |
| *Tank-*R | ATGATAGCGAGGTGAGGGTG |
| *Tnfaip3*-F | CAACAGAAGAGAGCCAGGGA |
| *Tnfaip3*-R | TGAGCAAGGGAACAGGAGAG |
| *Tnf-*F | ATGTGGCAAGAGATGGGGAA |
| *Tnf-*R | CTCACACCCCACATCTGTCT |

**Table S2: Antibodies used in flow cytometry**

| **Antibody** | **Source** | **Identifier** |
| --- | --- | --- |
| **CD274** (PD - L1) - FITC - Anti Human - Clone MIH1 -Isotype Mouse IgG1, kappa | BD Biosciences | Cat #: 558065 |
| **CD209** - PE - Anti Human – Clone  DCN46 – Isotype Mouse IgG 2b kappa | BD Biosciences | Cat #: 551265 |
| **HLA-DR** - APC - Anti Human - Clone  G46-6 (L243) -Isotype Mouse IgG 2a kappa | BD Biosciences | Cat #: 559866 |
| **CD80** - PE - Anti Human – Clone  L307.4 – Isotype Mouse IgG1 kappa | BD Biosciences | Cat #: 557227 |
| **CD86** - FITC - Anti Human - Clone  2331 (FUN-1) -Isotype Mouse IgG1 kappa | BD Biosciences | Cat #: 555657 |
| **CD83** - APC - Anti Human - Clone  HB15e – Isotype Mouse IgG1 kappa | BD Biosciences | Cat #: 551073 |

**Table S3: CBA Flex set used in the study**

| **CBA Flex Kit** | **Source** | **Identifier** |
| --- | --- | --- |
| Human IL-6 CBA Flex Set A7 | BD Biosciences | Cat #: 558276 |
| Human IL-8 CBA Flex Set A9 | BD Biosciences | Cat #: 558277 |
| Human IL-10 CBA Flex Set B7 | BD Biosciences | Cat #: 558274 |
| Human IL-12 p70 CBA Flex Set E5 | BD Biosciences | Cat #: 558283 |
| Human MCP-1 CBA Flex Set D8 | BD Biosciences | Cat #: 558287 |
| Human RANTES CBA Flex Set D4 | BD Biosciences | Cat #: 558324 |
| Human TNF CBA Flex Set C4 | BD Biosciences | Cat #: 560112 |
